# On the Competition Between Electron Autodetachment and Dissociation of Molecular Anions

**DOI:** 10.1007/s13361-019-02237-z

**Published:** 2019-05-28

**Authors:** Gerd Marowsky, Jürgen Troe, Albert A. Viggiano

**Affiliations:** 1 Laser-Laboratorium Göttingen, Hans-Adolf-Krebs-Weg 1, 37077 Göttingen, Germany; 2 Institut für Physikalische Chemie, Universität Göttingen, Tammannstrasse 6, 37077 Göttingen, Germany; 3 Max-Planck-Institut für Biophysikalische Chemie, Am Fassberg 11, 37077 Göttingen, Germany; 4 Air Force Research Laboratory, Space Vehicles Directorate, 3550 Aberdeen Avenue SE, Bldg 570, Kirtland Air Force Base, Albuquerque, NM 87117-5-776 USA

**Keywords:** Electron autodetachment, Anion dissociation, Rotational channel switching

## Abstract

We treat the competition between autodetachment of electrons and unimolecular dissociation of excited molecular anions as a rigid-/loose-activated complex multichannel reaction system. To start, the temperature and pressure dependences under thermal excitation conditions are represented in terms of falloff curves of separated single-channel processes within the framework of unimolecular reaction kinetics. Channel couplings, caused by collisional energy transfer and “rotational channel switching” due to angular momentum effects, are introduced afterward. The importance of angular momentum considerations is stressed in addition to the usual energy treatment. Non-thermal excitation conditions, such as typical for chemical activation and complex-forming bimolecular reactions, are considered as well. The dynamics of excited SF_6_^−^ anions serves as the principal example. Other anions such as CF_3_^−^ and POCl_3_^−^ are also discussed.

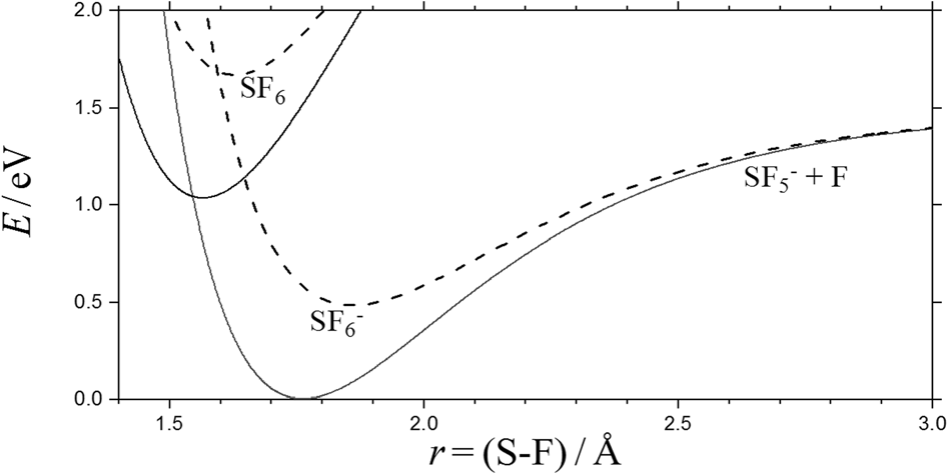

## Introduction

Vibrationally excited molecular anions may undergo a variety of processes such as dissociation to anionic and neutral fragments, autodetachment of electrons, radiative stabilization, and collisional deactivation (or activation). The competition between these channels is governed by the energy *E* and the rotational state of the anion (the latter symbolically characterized by an angular momentum quantum number *J*). While the influence of the energy is always taken into account, angular momentum effects are often neglected. As the overall reaction represents a multichannel system, channel coupling effects also have to be accounted for. The present article intends to illustrate the competition between the various channels using thermally excited SF_6_^−^ anions as the main example. Other anions are considered as well. Finally, non-thermal excitation conditions are discussed with respect to angular momentum effects.

At sufficiently high energies, vibrationally excited anions SF_6_^−^* may react by

1 $$ {{\mathrm{SF}}_6}^{-\ast}\kern0.5em \to {{\mathrm{SF}}_5}^{-}+\mathrm{F} $$

2 $$ {{\mathrm{SF}}_6}^{-\ast}\kern0.5em \to {\mathrm{SF}}_6+{\mathrm{e}}^{-} $$

3 $$ {{\mathrm{SF}}_6}^{-\ast}\kern0.5em \to {{\mathrm{SF}}_6}^{-}+\mathrm{h}\nu $$

4 $$ {{\mathrm{SF}}_6}^{-\ast }+\mathrm{M}\kern0.5em \to {{\mathrm{SF}}_6}^{-}+\mathrm{M} $$

5 $$ {{\mathrm{SF}}_6}^{-}+\mathrm{M}\kern0.5em \to {{\mathrm{SF}}_6}^{-\ast }+\mathrm{M} $$

**Figure 1:**
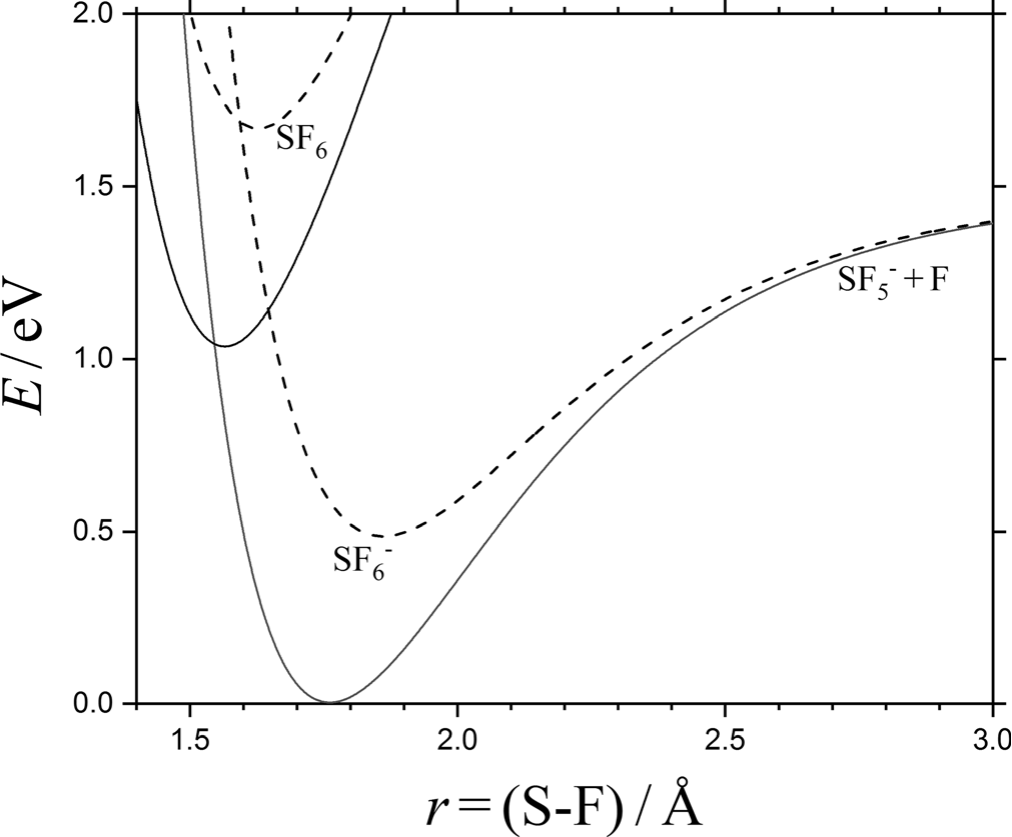
Potential curves for SF_6_ → SF_5_ + F and SF_6_^−^ → SF_5_^−^ + F at *J* = 0 (full lines) and *J* = 250 (dashed lines) (Morse potentials with exponentially damped centrifugal energies and data from [4, 7], leading to E_0,1_(*J* = 0) ≈ 1.44 eV and E_0,1_(*J* = 250) ≈ 0.90 eV for dissociation of SF_6_^−^, and E_0,2_(*J* = 0) ≈ *E*_0,2_(*J* = 250) ≈ 1.03 eV for electron detachment from SF_6_^−^)

At even higher energies, the additional dissociation

6 $$ {{\mathrm{SF}}_6}^{-\ast}\kern0.5em \to {\mathrm{SF}}_5+{\mathrm{F}}^{-} $$

may be included. Reaction (1) corresponds to a simple bond fission with a loose activated complex (AC) which is located at the centrifugal maximum of an ion-induced dipole potential (plus some valence contributions, see, e.g., [1, 2]). In contrast to Reaction (1), Reaction (2) effectively involves a rigid AC, located at the crossing of the SF_5_^−^-F and SF_5_-F potential curves [3–6]. Figure 1 illustrates this crossing for the non-rotating and rotating SF_6_^−^/SF_6_ system in comparison to the potential of the dissociating anion SF_6_^−^. The crossing in the SF_6_^−^- system probably involves a small energy barrier, but even without that barrier, the crossing occurs at a more compact nuclear configuration of SF_6_^−^ than that relevant for Reaction (1). Considering the nuclear motion only, the system then is of rigid-AC/loose-AC character (one has to note, however, that nuclear and electronic motions in this description are separated which is an essential element of the “kinetic modeling approach” as justified later on).

One of the consequences of the rigid-AC/loose-AC character is markedly different *J* dependences of the channel threshold energies *E*_0,*i*_ (the subscript *i* = 1 corresponds to the dissociation channel (1) while *i* = 2 corresponds to the detachment channel (2)). This may even lead to “rotational channel switching” [8, 9] of channels (1) and (2). While *E*_0,1_(*J* = 0) is larger than *E*_0,2_(*J* = 0), at some value of *J* (denoted by *J*_sw_), the ordering of the *E*_0,i_ may change from *E*_0,1_(*J*) > *E*_0,2_(*J*) for *J* < *J*_sw_ to *E*_0,1_(*J*) < *E*_0,2_(*J*) for *J* > *J*_sw_. As this is also of relevance for non-thermal conditions, this effect will be further explored below.

The branching fraction of the reaction

7 $$ \mathrm{R}\left({{\mathrm{SF}}_5}^{-}\right)=\left[{{\mathrm{SF}}_5}^{-}\right]/\left(\left[{{\mathrm{SF}}_5}^{-}\right]+\left[{{\mathrm{SF}}_6}^{-}\right]\right) $$

may be derived from a master equation simulation of the multilevel system symbolized by Reactions (1)–(6). This simulation leads to “falloff curves” (i.e., dependences of the rate constants at fixed temperature *T* on the bath gas concentration [M]) of both the overall thermal dissociation rate constants *k*_dis_ (defined by the rate law *d*[SF_5_^−^]/*dt* = *k*_dis_ [SF_6_^−^]) and the overall detachment rate constants *k*_det_ (defined by the rate law *d*[e^−^]/*dt* = *k*_det_ [SF_6_^−^]). First, these falloff curves may be calculated for “separated channels” (e.g., with the channels (1), (4), and (5) for *k*_dis_ and with the channels (2), (4), and (5) for *k*_det_). Afterward, proper modeling requires channel coupling effects to be taken into account [10]. It is emphasized that the SF_6_^−^- system is not unique in this regard; other anion fragmentation processes will behave in an analogous way.

## Falloff Curves for Separated Electron Detachment and Dissociation Processes of SF_6_^−^

Falloff curves for non-dissociative electron attachment to SF_6_ (in the presence and absence of radiative stabilization (3)) have been elaborated within the “kinetic modeling approach” of [11]. The rate coefficients *k*_at_ were determined for equal electron and bath gas temperatures *T* between 200 and 1400 K and for bath gas concentration [N_2_] between 10^10^ and 10^20^ cm^−3^. Like other falloff curves, these can be represented in the form [12]

8 $$ k/{k}_{\infty }=\left[x/\left(1+x\right)\right]\ F(x) $$

with rate coefficients *k*, limiting high-pressure rate coefficients *k*_∞_, limiting low-pressure rate coefficients *k*_0_ (being proportional to [N_2_] and of the same dimension as *k*_∞_), *x* = *k*_0/_*k*_∞_, and “broadening factors” *F*(*x*) approximated by

9 $$ \log\ F(x)\approx \left\{\log\ {F}_{\mathrm{cent}}\right\}/\left\{1+{\left[\left(\log\ x\right)/N\right]}^2\right\} $$

where *F*_cent_ = *F*(*x* = 1) and *N* = 0.75–1.27 log *F*_cent_ (where log = ^10^log). Taking advantage of the modeling of *k*_at,0_, *k*_at,∞_, and *F*_at,cent_ for electron attachment of [11] and inserting these values for *k*_0_, *k*_∞_, and *F*_cent_ into Eq. (8), *k*_at_ is obtained. It then can be converted into thermal rate coefficients for detachment *k*_det_, employing the corresponding equilibrium constant

10 $$ {K}_{\mathrm{det}}={k}_{\mathrm{det}}/{k}_{\mathrm{at}}={\left(\left[{e}^{-}\right]\left[{\mathrm{SF}}_6\right]/\left[{{\mathrm{SF}}_6}^{-}\right]\right)}_{\mathrm{eq}} $$

The following parameters were calculated for the falloff curves of *k*_at_ (without radiative stabilization (3)): *k*_at,0_ ≈ [N_2_] 2.5 × 10^−18^ exp(− *T*/80 K) [1 + 3.5 × 10^−22^ (*T*/K)^7^] cm^6^ s^−1^, *k*_at,∞_ ≈ 2.2 × 10^−7^ (*T*/500 K)^-0.35^ cm^3^ s^−1^, and *F*_at,cent_ ≈ exp(− *T*/520 K) [11, 13].

Since the publication of [11, 13], the electron affinity EA of SF_6_ has been disputed [4, 7, 14–17]. As *K*_det_ and *k*_det_ both include a factor exp(− EA/*k*_B_*T*), the value of EA is of primary importance for these two quantities. In addition to EA, also the vibrational partition function *Q*_vib_ (SF_6_^−^) had to be modified [7], because marked anharmonicities of the vibrations of SF_6_^−^ were discovered in [4]. These refinements influence not only *K*_det_, *k*_det_, and *k*_at_ but also the falloff curves for dissociating SF_6_^−^. This is illustrated in the following.

Falloff curves for *k*_dis_, i.e., for the dissociation of SF_6_^−^ to SF_5_^−^ + F, are also represented in the form of Eq. (8). In this case, it appears appropriate to start with the limiting high-pressure rate coefficients *k*_rec,∞_ ≈ 2.15 × 10^−10^ cm^3^ s^−1^ for combination of an ion with a neutral species in a charge-induced dipole potential (see [13]; *k*_rec,∞_ here is assumed to be independent of the temperature). With the corresponding equilibrium constant,

11 $$ {K}_{\mathrm{dis}}\approx {k}_{\mathrm{dis}}/{k}_{\mathrm{rec}}={\left(\left[{{\mathrm{SF}}_5}^{-}\right]\left[\mathrm{F}\right]/\left[{{\mathrm{SF}}_6}^{-}\right]\right)}_{\mathrm{eq}} $$

this leads to *k*_dis,∞_. On the other hand, the limiting low-pressure rate coefficient *k*_dis,0_ can directly be calculated from the unimolecular rate theory as elaborated in [12]. Analogous to *K*_det_ and *k*_det_, both *K*_dis_ and *k*_dis_ include a factor exp[−EA/k_B_*T*]. In addition, however, they include the factor exp[−∆E_0_/k_B_T] where ∆E_0_ corresponds to the energy difference between SF_6_ + e^−^ and SF_5_^−^ + F at 0 K (being 0.41 eV [7]). Furthermore, *K*_dis_ and *k*_dis_ include the strongly anharmonic vibrational partition function *Q*_vib_(SF_6_^−^). Analogous to the dispute about the EA of SF_6_, the energy difference ∆E_0_ has multiple values in the literature (see, e.g., [3, 7, 13, 14, 18–23]). (The dissociation channel (6) of SF_6_^−^*requires higher energies than SF_5_^−^ formation [22] and, therefore, is not further considered here.) In view of the difficulties with EA, ∆E_0_, and *Q*_vib_ (SF_6_^−^), it appears important to analyze to what extent the modeled rate constants become independent of these difficulties, because some of the uncertainties compensate each other.

The largest uncertainties encountered in the modeling of *k*_dis,0_ can be estimated within the formulation of the unimolecular rate theory described in [12]. *k*_dis,0_ contains a factor *ρ*_vib,h_ (EA + ∆E_0_) *F*_anh_/*Q*_vib_ for SF_6_^−^, where *ρ*_vib,h_ (EA + ∆E_0_) denotes the harmonic vibrational density of states and *F*_anh_ is an anharmonicity factor. The anharmonicity contributions in *Q*_vib_ and the factor *F*_anh_ in part compensate each other. However, the anharmonicity in *Q*_vib_ has been essential in the third-law evaluation by [7] of the experimental ratio *k*_*d*et_/*k*_*a*t_ = *K*_det_, leading to the electron affinity EA = 1.03(± 0.05) eV. It should be mentioned that this value was supported by the most detailed quantum chemical calculations of [15]. In the modeling of *k*_dis,0_, besides EA + ∆E_0_ and the ratio *ρ*_vib,h_ (EA + ∆E_0_) *F*_anh_/*Q*_vib_, the average energy <∆*E*_coll_> transferred per collision between SF_6_^−^* and M remains an uncertain parameter. Keeping in mind these uncertainties and leaving a fine-tuning of *k*_dis,0_ to the comparison with the experiments, we model *k*_dis,0_ with the harmonic frequencies of SF_6_^−^ from [24] (such as given in [13]), EA = 1.03 eV from [7], a total collisional energy transfer frequency approximated by the Langevin collision frequency *Z* = 6.37 × 10^−10^ cm^3^ s^−1^ (for collisions between SF_6_^−*^ and N_2_ [14]) and <∆*E*_coll_>/hc ≈ −200 cm^−1^ [25, 26]. This leads to

12 $$ {k}_{\mathrm{dis},0}\approx \left[{\mathrm{N}}_2\right]\ 4.3\ \mathrm{x}\ {10}^{-3}{\left(T/650\ \mathrm{K}\right)}^{\hbox{--} 11.6}\exp\ \left[-\left(\mathrm{EA}+\Delta {\mathrm{E}}_0\right)/{k}_{\mathrm{B}}T\right]\ {\mathrm{cm}}^3{\mathrm{s}}^{-1} $$

While *k*_dis,0_ (*T*) relies on modeling, *k*_det,0_ directly follows from the experimental *k*_at,0_ [7] and the revised *K*_det_ from [7], one obtains

13 $$ {k}_{\det, 0}\approx \left[{\mathrm{N}}_2\right]\ 3.4\ \mathrm{x}\ {10}^{-5}{\left(T/650\ \mathrm{K}\right)}^{-8.9}\exp\ \left[-\mathrm{EA}/{\mathrm{k}}_{\mathrm{B}}T\right]\ {\mathrm{cm}}^3{\mathrm{s}}^{-1} $$

**Figure 2:**
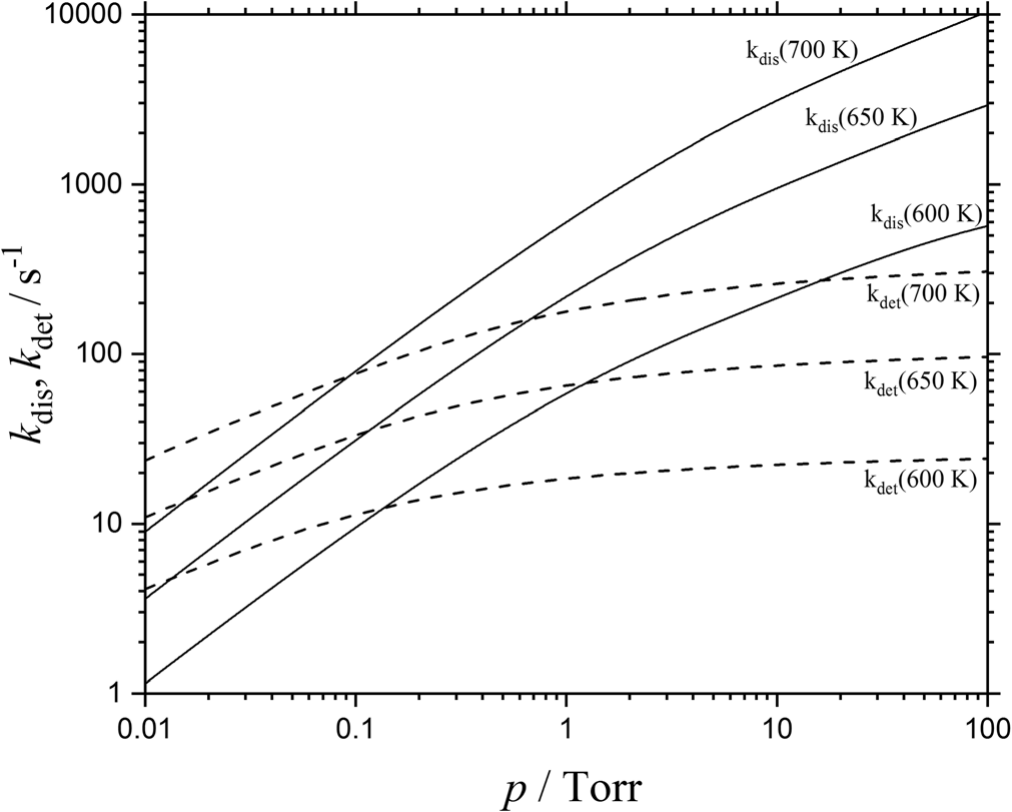
Falloff curves for autodetachment of electrons (dashed curves) and dissociation (full curves) of thermally excited SF_6_^−^ anions at 600, 650, and 700 K

**Figure 3:**
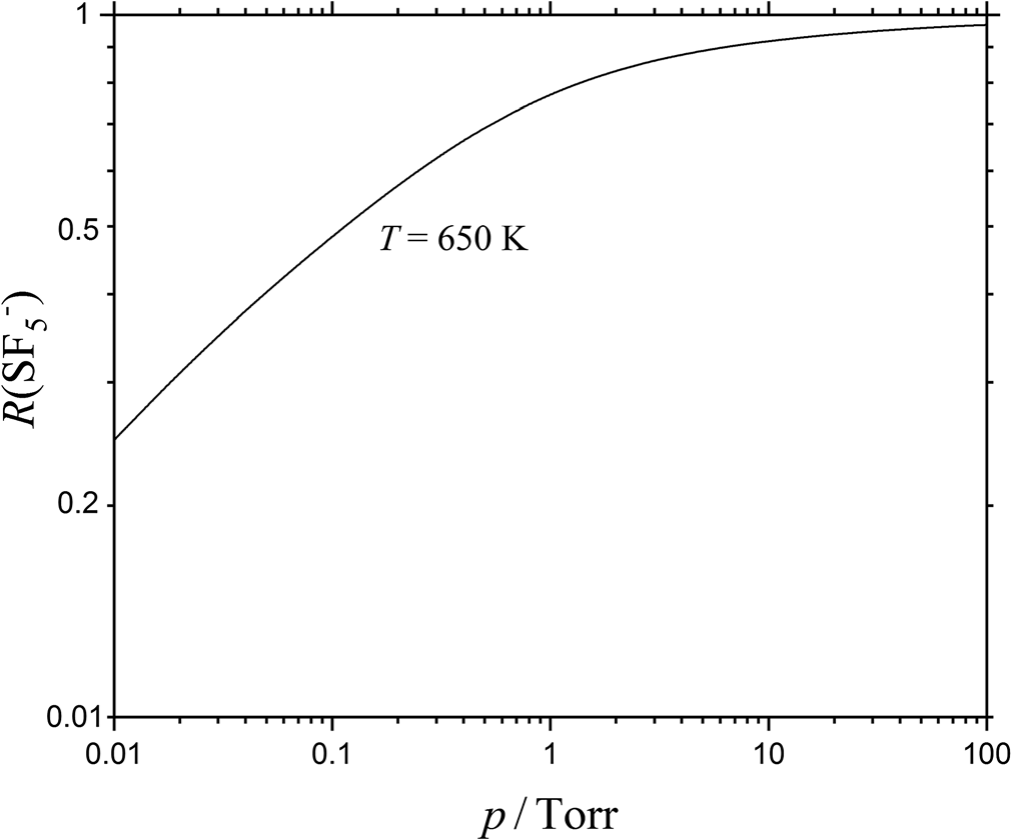
Branching fractions *R*(SF_5_^−^) = [SF_5_^-^]/([SF_5_^−^] + [SF_6_^−^]) = *k*_dis_/(*k*_dis_ + *k*_det_) for reaction of thermally excited SF_6_^−^ anions at 650 K (with *k*_dis_ and *k*_det_ from Figure 2)

Around 650 K, where measurements of the branching fraction *R*(SF_5_^−^) are available [13, 14, 27, 28], obviously *k*_det,0_ is much larger than *k*_dis,0_, i.e., *k*_det,0_ > *k*_dis,0_. This is in contrast to *k*_dis,∞_ and *k*_det,∞_ where the former is given by

14 $$ {k}_{\mathrm{dis},\infty}\approx 1.5\ \mathrm{x}\ {10}^{15}{\left(\mathrm{T}/650\ \mathrm{K}\right)}^{-1}\exp\ \left[-\left(\mathrm{EA}+{\Delta \mathrm{E}}_0\right)/{\mathrm{k}}_{\mathrm{B}}\mathrm{T}\right]\ {\mathrm{s}}^{-1} $$

while the latter amounts to

15 $$ {k}_{\det, \infty}\approx 1.1\ \mathrm{x}\ {10}^{10}{\left(T/650\ \mathrm{K}\right)}^{-1.4}\exp\ \left[-\mathrm{EA}/{k}_{\mathrm{B}}T\right]\ {\mathrm{s}}^{-1} $$

such that *k*_dis,∞_ > *k*_det,∞_. The comparison of the pre-exponential factors of Eqs. (14) and (15) classifies detachment as an effectively rigid-AC process and supports the view of the “kinetic modeling approach” given in the “Introduction.” On the other hand, dissociation is clearly a loose-AC bond fission reaction. The observation of *k*_dis,0_ < *k*_det,0_ and *k*_dis,∞_ > *k*_det,∞_ (near 650 K) indicates that there must be a crossing of the two falloff curves at some [N_2_] (denoted by [N_2_]_*x*_ or by the corresponding bath gas pressure *p*_*x*_). In order to locate *p*_*x*_, we also need *F*_cent_ which, for simplicity, we use in the form *F*_dis,cent_ ≈ *F*_at,cent_ as calculated in [11]. Figure 2 illustrates pairs of falloff curves for 600, 650, and 700 K. The curves cross near [N_2_]_*x*_ ≈ 1.5 × 10^15^ cm^−3^ (corresponding to *p*_*x*_ ≈ 0.1 Torr). At this pressure, dissociation is close to its low-pressure limit while detachment is closer to its high-pressure limit. Figure 3 shows the corresponding branching fraction *R*(SF_5_^−^) for *T* = 650 K, being constructed with *R*(SF_5_^−^) = *k*_dis_/(*k*_dis_ + *k*_det_) from Figure 2 (it should be mentioned that Figure 3 is consistent with Figures 8 and 9 of [14]). As the exponential factor exp[− ∆E_0_/k_B_*T*] dominates *R*(SF_5_^−^), while other not so well-known contributions have only weaker temperature dependences, the evaluation of the temperature dependence of *R*(SF_5_^−^) provides safe access to ∆E_0_. This formed the basis for the fit of ∆E_0_ ≈ 0.41 eV in [7, 29]. However, channel coupling effects were neglected so far. Therefore, one has to make sure that rotational channel switching and the related multichannel coupling effects do not matter too much. In the following section, we explore to what extent the rigid-AC/loose-AC multichannel character of the system requires multichannel coupling corrections.

## Rotational Channel Switching and Multichannel Coupling Effects in SF_6_^−^

The foregoing section provided falloff curves for separated electron detachment and dissociation of thermally excited SF_6_^−^. It illustrated that electron detachment in the language of “kinetic modeling” effectively proceeds as a rigid-AC process whereas dissociation is a loose-AC process. In this situation, rotational channel switching, such as described in the “Introduction,” modifies the branching fractions which—so far—were calculated assuming separated, single-channel, falloff curves.

The rigid AC of the electron detachment process is located at the nuclear configuration where the potential curves of SF_5_-F and (SF_5_-F)^−^ cross (see Figure 1). This crossing happens at an S-F distance *r*_*x*_ ≈ 1.58 Å which corresponds to a structure with an effective rotational constant B_e_ (*r*_e_/*r*_*x*_)^2^ ≈ B_e_ (B_e_ is the rotational constant, being 0.0907 cm^−1^ for SF_6_ and 0.0750 cm^−1^ for SF_6_^−^, while *r*_e_ ≈ 1.56 Å for SF_6_ and 1.76 Å for SF_6_^−^). The threshold energy E_det,0_ (*J*) for rotating SF_6_^−^ then roughly increases as

16 $$ {\mathrm{E}}_{0,\det }(J)\approx {\mathrm{E}}_{0,\det}\left(J=0\right)+{\mathrm{B}}_{\mathrm{e}}h\ c\ J\ \left(J+1\right) $$

where

17 $$ {\mathrm{E}}_{0,\det}\left(J=0\right)\approx \mathrm{EA}+5.2\ \mathrm{meV} $$

(a barrier of about 5.2 meV in [5] was fitted with the help of the low-temperature experiments of [6]; however, this value is only of little relevance for the estimate of *J*_sw_). The threshold energies *E*_0,dis_ (*J*) correspond to the centrifugal barriers in the (SF_5_^−^-F) potential and can be estimated for an ion-induced dipole potential as shown in [1]. As the second term of Eq. (17) and the extra energy due to the centrifugal maxima in the dissociation process in excess of the energy EA + ∆E_0_ are both small compared to ∆E_0_, they are neglected here. The switching value *J*_sw_ then follows from the relationship

18 $$ {\mathrm{B}}_{\mathrm{e}}h\ c{J}_{\mathrm{sw}}\left({J}_{\mathrm{sw}}+1\right)\approx \Delta {\mathrm{E}}_0 $$

With ∆E_0_ ≈ 0.41 eV, this leads to

19 $$ {\mathrm{J}}_{\mathrm{sw}}\approx 191 $$

For *J* > *J*_sw_, E_0,det_ (*J*) becomes larger than E_0,dis_ (*J*), i.e., rotational channel switching occurs and rotationally hot SF_6_^−^ has a smaller threshold energy for dissociation than for electron detachment.

Rotational channel switching is the dominant cause for channel coupling in rigid-AC/loose-AC, two-channel, reaction systems [10]. Branching fractions *R*_1_ for the energetically less favorable channel (at *J* = 0; *R*_1_ corresponds to the energetically less favorable channel) are defined by *R*_1_ = *k*_1_/(*k*_1_ + *k*_2_). At a given temperature, *R*_1_ varies with the bath gas concentration [M]. It increases from a limiting low-pressure value of *R*_1,0_ to a limiting high-pressure value of *R*_1,∞_. This increase can be represented in approximate form by

20 $$ {R}_1\approx {R}_{1,0}+\left({R}_{1,\infty }-{R}_{1,0}\right)\ x/\left(x+1\right) $$

where *x* = [M]/[M]_cent_ ([M]_cent_ denotes that [M] for which *x* = 1 in Eq. (8)). The limiting low-pressure value *R*_1,0_ is related to *J*_sw_ by

21 $$ {R}_{1,0}\approx \exp\ \left[-{\mathrm{B}}_{\mathrm{e}}\mathrm{hc}\ {J}_{\mathrm{sw}}\left({J}_{\mathrm{sw}}+1\right)/{\mathrm{k}}_{\mathrm{B}}T\right] $$

For SF_6_^−^, this leads to *R*_*1*,0_ ≈ exp(− 4760 K/*T*). Channel coupling effects, therefore, only become important at very high temperatures for the present case. Branching fractions *R*(SF_5_^−^), corresponding to *R*_1_, at lower temperatures, thus can be calculated with the separated channel rate constants *k*_det_([M]) and *k*_dis_([M]) (and [M]_cent_ ≈ [N_2_]_*x*_ as shown in Figure 3) while channel coupling effects remain negligible. The results of the previous section (as illustrated by Figure 3), therefore, were not “contaminated” by rotational channel switching and channel coupling effects.

## Non-thermal Activation Conditions

It has to be emphasized that the described analysis of channel coupling effects in terms of Eq. (20) applies to thermal energy and angular momentum distributions only. In many experiments, however, the anions are produced with non-thermal distributions. For example, dissociative electron attachment (DEA) experiments start with non-thermal distributions of the states of the anions. These relax toward thermal distributions only in the presence of collisions. DEA then behaves as a “chemical activation system.” The corresponding relaxation of the branching fractions *R*(SF_5_^−^) toward their equilibrium values has been followed experimentally in [14]. For the time during the relaxation, master equation simulations have to describe the competition between the reaction steps (1)–(3) and the collision processes (4) and (5). The yields of the corresponding chemical or photochemical activation systems as a function of the primary excitation energy and the bath gas pressure have been modeled in [27]. The results can directly be applied to DEA. Meanwhile, the uncertainty in the value of <∆*E*_coll_> for collisional energy transfer and, in particular, of the change of the angular momentum distribution during the collisional relaxation limits the accuracy of the simulation. Further work is required to analyze the consequences of rotational channel switching under non-thermal activation conditions which are certainly different from those of the thermal excitation analyzed here. Finally, the analogy of the chemical activation situation to the pressure and temperature dependence of complex-forming bimolecular reactions should be stressed, such that the approximate expressions for yields from the corresponding treatment may become helpful [28].

Apart from rotational channel switching in rigid-AC/loose-AC multichannel systems, also “vibrational channel switching,” particularly under non-thermal excitation conditions, is of importance [9]. The specific rate constants *k*_dis_(*E*,*J*) for fixed *J* at some energy *E*_sw_ then cross the corresponding *k*_det_(*E*,*J*). This was illustrated, e.g., for DEA of SF_6_^−^ at *J* = 0 in Figure 5 of [13]. Under thermal excitation conditions, this effect is responsible for the markedly different pre-exponential factors of *k*_dis,∞_(*T*) and *k*_det,∞_(*T*) in Eqs. (14) and (15). Under non-thermal excitation conditions and in the absence of collisions, the differences of the *k*(*E*,*J*) will cause quite different time dependences of the decaying anions. Energy and angular momentum as well as channel switching effects then will all have to be taken into account. Oversimplification of the multichannel character of the process and its energy and angular momentum dependence may have been the reason for different interpretations of experiments (possibly also for the different values derived for EA of SF_6_ in [4, 7, 14–17]).

## Systems with Loose-AC/Rigid-AC and Rigid-AC/Rigid-AC Channels

Analogous to the SF_6_^−^ example, one should inspect rotational channel switching effects in other DEA systems. First, we consider the CF_3_^−^ example where

22 $$ {{\mathrm{CF}}_3}^{-}\ast \to {\mathrm{CF}}_3+{\mathrm{e}}^{-} $$

23 $$ {{\mathrm{CF}}_3}^{-}\ast \to {\mathrm{F}}^{-}+{\mathrm{CF}}_2 $$

compete. With an electron affinity of EA = 1.82 (± 0.05) eV for CF_3_ [30] and an energy difference ∆E_0_ = 0.22 (± 0.02) eV [31], this system according to Eq. (18) has a smaller *J*_sw_ than SF_6_^−^. The crossing between the (CF_2_-F) and (CF_2_-F)^−^ potential curves here takes place at *r*_*x*_ ≈ *r*_e_ [32], such that *J*_sw_ ≈ 70 (with B_e_ ≈ 0.360 cm^1^). This confirms again a loose-AC/rigid-AC character of the system. Experimental studies of the DEA to CF_3_ [32, 33] so far have only been concerned with the chemical activation regime of the process, and rotational channel switching effects were not yet considered. If the process would have been followed over the relaxation period from chemical activation to thermal distributions, the branching fraction would have been characterized by Eq. (20) with *R*_1,0_ ≈ exp(− 2570 K/*T*). Obviously, this would have been relevant for temperatures which were beyond those considered so far. However, as emphasized above, channel switching effects are important as well during the relaxation stage typically achieved in DEA experiments.

Multichannel coupling effects caused by rotational channel switching are ubiquitous, e.g., in DEA to other fluorocarbon radicals [34], in DEA to CF_3_Br [33], or in DEA to POCl_3_ [35–37]. The latter system could be affected by rotational channel switching in particular, as small values of ∆E_0_ are observed (∆E_0_ ≈ 0 for the production of POCl_2_^−^ + Cl and ∆E_0_ = 0.11 eV for the production of POCl_2_ + Cl^−^). The preliminary modeling with a chemical activation scheme here was successful under the assumption of a loose AC for the POCl_2_ + Cl^−^ channel while a more rigid AC was found for the POCl_2_^−^ + Cl channel. The presence of several competing channels with different individual *J*_sw_ further complicates the analysis. In this case, branching fractions under thermal and non-thermal conditions may take advantage of the multichannel codes elaborated in [10].

One observation from the analysis of the experiments on the POCl_3_ system in [37] deserves further attention. Assuming a loose-AC character for all dissociation channels, “rigidity factors” *f*_rigid_ in that analysis were fitted. These factors account for an anisotropy of the potential beyond the isotropy of the dominant ion-induced dipole potential between the dissociation fragments. This fitting in [37] led to markedly smaller values of *f*_rigid_ for the nearly thermoneutral POCl_2_^−^ + Cl channel than for the endothermic POCl_2_ + Cl^−^ channel. This observation may suggest that the former channel involves some intermediate energy barrier. This might signal rigid-AC channel behavior of this dissociation channel. Multichannel coupling effects under thermal conditions for rigid-AC/rigid-AC then would be characterized by Eq. (20) with

24 $$ {R}_{1,0}\approx \exp\ \left(-\Delta  {E}_0/\gamma \right) $$

where *γ* denotes the average energy transferred per up collision (related to the total <∆*E*_coll_> by <∆*E*_coll_>/hc = *γ* − *α* where *γ* ≈ *α*k_B_*T*/(*α* + k_B_*T*; *α* and *γ* traditionally are given in cm^−1^) and *α* is the average energy transferred per down collision). In this case, instead of rotational channel switching, collisional processes would be responsible for multichannel coupling effects.

## Conclusions

The present article characterizes the competition between electron autodetachment and fragmentation of vibrationally excited molecular anions in the language of chemical kinetics. The main conclusion consists in the statement that autodetachment of electrons effectively corresponds to a rigid-activated complex process, while fragmentations mostly have loose activated complexes (although sometimes the latter also may be governed by rigid-activated complexes). A rigid-AC/loose-AC character of the reaction gives rise to rotational channel switching where energetically less favorable reaction channels dominate over energetically more favorable channels when the ion rotates rapidly. In the presence of collisions, also multichannel coupling effects have to be taken into account. The branching fractions under thermal excitation conditions can be represented approximately by Eqs. (18), (20), and (21). The importance of energy and angular momentum effects under non-thermal, chemical-activation type, excitation conditions is stressed as well.
